# Bridging farmers’ perceptions and laboratory science: wheat bran quality assessment in smallholder dairy systems: technical note

**DOI:** 10.5713/ab.24.0810

**Published:** 2025-04-04

**Authors:** Alemayehu Tadesse, Yayneshet Tesfay, Veerle Fievez

**Affiliations:** 1 Department of Animal Sciences and Aquatic Ecology, Faculty of Bioscience Engineering, Ghent University, Gent, Belgium; 2 Department of Animal, Rangeland and Wildlife Sciences, Mekelle University, Mekelle, Ethiopia; 3 Independent Researcher

**Keywords:** Nutritive Value, Particle Size, Water Holding Capacity, Wheat Bran

## Abstract

This study was conducted to evaluate wheat bran (WB) quality characteristics based on particle size distribution and to assess how farmers’ rankings align with laboratory results. Thirty smallholder dairy farmers qualitatively scored five WB types from five major wheat flour processing factories in Tigray, Ethiopia, on a scale of 1 to 4 for water holding capacity (WHC) and nutritive value (1 = poor to 4 = excellent), as well as the risk of digestive disorders (1 = high to 4 = very low). Laboratory analyses included physical parameters, chemical analysis, and *in vitro* digestibility. The geometric mean particle size of WB ranged between 908 to 1,103 μm, and the WHC between 2.15 to 2.90 mL/g. Farmers’ scores for nutritive value correlated positively with crude protein (r_spearman’s_ = 0.347; p<0.05) and effective rumen degradability of crude protein (r_spearman’s_ = 0.291; p<0.05), and negatively with particle size (r_spearman’s_ = −0.553; p<0.05). Scores for WHC positively correlated with particle size (r_spearman’s_ = 0.526; p<0.05). The present findings revealed that particle size distribution is the predominant qualitative selection criteria for farmers (e.g., on the market) to assess WB quality, and this qualitative appreciation is to some extent related to chemical characteristics and rumen degradability.

## INTRODUCTION

Wheat, the primary cereal crop used as an industrial raw material by flour and food-based factories in Ethiopia, generates an estimated annual production of approximately 1.9 million tonnes of wheat bran as its main by-product from flour milling [1]. Wheat bran is commonly used in Ethiopian smallholder dairy farms to supplement the basal diet. Its role in supporting milk production is evidenced by a study [2] showing that high-producing cows received higher allocations. Despite being Ethiopia’s top traded livestock feed, standardized quality parameters for marketing wheat bran are absent. Dairy farmers in northern Ethiopia qualitatively assess wheat bran based on particle size, attempting to judge nutritive value (finer particle size presumed to have a higher endosperm-to-bran ratio and hence, higher nutritive value), hydration properties (e.g., water holding capacity; WHC), with products with enhanced WHC indicating higher palatability) and risks of digestive disorders (e.g., bloating and acidosis associated with extremely fine particles). Former research indicated that the evaluation for physical attributes primarily was based on the particle size distribution and related wheat bran particle size to its hydration properties [3,4]. Understanding farmers’ feed quality perceptions is vital for optimizing livestock productivity as they shape feed choices, management practices, and adoption of innovations [5]. Complementarity between feed value, based on farmers’ expert knowledge, and laboratory indicators has been documented for indigenous fodder trees and shrubs [6] and exotic multipurpose forage trees [5]. However, the characterization of wheat bran particle size distribution has received little attention. Besides, there is limited or no information on the alignment between farmers’ qualitative assessments and laboratory standards for wheat bran, which is crucial for bridging the gap between subjective and objective feed quality evaluation to improve livestock nutrition in practice. This study aims to evaluate wheat bran quality characteristics based on particle size distribution and to assess the relationship between farmers’ assessment and laboratory analyses of wheat bran quality parameters.

## MATERIALS AND METHODS

A total of 30 smallholder dairy farmers (10 from each of three districts) with prior experience in feeding wheat bran to dairy cows were randomly selected from three major dairy-producing districts of Tigray region—Hagereselam (highland agroecology), Agula (midland agroecology), and Alamata (lowland agroecology)—recognized for their dairy production potential and long-standing experience. Five types of wheat bran were collected from five major wheat flour processing factories in Tigray (North Ethiopia). The ethical commission of the Institute for Agricultural and Fisheries Research (ILVO, Belgium; file number EC2015-257) approved the fistulation of the animals from which the rumen fluid was collected for the *in vitro* rumen incubation experiment.

### Particle size distribution and mean particle size

The particle size distribution of raw (ungrounded) wheat bran was determined by sieving 20 g of sample through a series of sieves (4,000, 2,800, 2,000, 1,000, 500, 212, 100, 75, and 50 μm) on a vibratory sieve shaker at 90 RPM for 30 min [3]. The geometric mean diameter (D50) and geometric standard deviation (S_gw_) were calculated according to Wilcox et al [7].

### Physical parameters

The physical parameters (swelling capacity [SC], WHC and bulk density [BD]) of raw (ungrounded) wheat bran were determined as follows. The SC was determined according to the method described by Jacobs et al [3], where 0.5 g of wheat bran was immersed in 5.0 mL of distilled water in a 10-mL graduated cylinder. After 60 minutes of hydration, the final volume of the swollen bran was recorded to quantify SC. The WHC was determined following filtration method [8]. The 1 g wheat bran sample was soaked in 100 mL distilled water for 24 h. After soaking, the sample was filtered through a fritted glass crucible (porosity 2) under vacuum pump. The wet sample was weighed post-filtration, with the retained water quantified as WHC. BD was measured according to Onipe et al [4] using a clean and dry 100-mL graduated cylinder. The cylinder was weighed, filled with wheat bran to the 50 mL mark, and swirled for 15 seconds. The volume was recorded, and the combined weight of the cylinder and contents was measured. BD was calculated as the sample weight (g) divided by the sample volume (mL).

### Chemical analysis

Wheat bran samples ground and particles retained between 1 and 0.212 mm sieves underwent chemical and *in vitro* incubation analyses. Dry matter (DM), ash, nitrogen, and ether extract (EE) were determined according to AOAC [9]. Neutral detergent fiber (aNDF_OM_) was assayed using heat-stable amylase and sulphite and expressed exclusive of residual ash. Acid detergent fiber (ADF) was measured with the ANKOM^200^ fiber analyzer (ANKOM Technology, Macedon, NY, USA) following Van Soest et al [10]. The brief descriptions of these analysis were given in Tadesse et al [2]. Non-fiber carbohydrates (NFC, g/kg DM) were calculated as 1,000 – (aNDF_OM_ [g/kg DM] + CP [g/kg DM] + EE [g/kg DM] + Ash [g/kg DM]).

### *In vitro* incubation

Wheat bran samples were incubated in an *in vitro* rumen simulation for 2, 4, 6, 10, 24, 48, and 72 hours to assess DM and CP degradability as described in the procedures by Lima-Orozco et al [11]. The DM and CP disappearance data were modeled using Ørskov and McDonald [12] exponential model. Effective rumen degradability (ERD, g/g) of DM and CP was calculated using a fractional passage rate (kp) of 0.03/h [12]. Detailed descriptions for chemical analysis and *in vitro* incubation were described in Tadesse et al [2].

### Dairy farmers’ qualitative assessment of wheat bran

Farmers were trained on evaluation criteria descriptors, and to avoid bias, wheat bran types were coded, with each farmer conducting assessments independently. Farmers evaluated the wheat bran types on a scale of 1 to 4 for nutritive value and WHC (1: poor, 2: fair, 3: good, 4: excellent) and for the risk of digestive disorder (1: high, 2: moderate, 3: low, 4: very low). The ranking index (RI) for the studied five wheat bran types was calculated as per Gizaw et al [13]: RI = (Σ R_n_ x C_1_ + R_n-1_ x C_2_…. + R_1_ x C_n_) / (Σ C_1_ + C_2_…+ C_n_); where, R_n_ = value given for the least ranked level (example if the least rank is 5, then R_n_ = 5, R_n–1_ = 4, R_1_ = 1). C_n_ = counts of the least ranked level (in the above example, the count of the 5^th^ rank = C_n_, and the count of the 1^st^ rank = C_1_).

### Statistical analysis

The statistical analyses were performed using JMP SAS version 16 (SAS Institute, Cary, NC, USA). Wilcoxon rank sum test was used for the ranking of quality attributes between wheat bran types. Spearman’s rank correlation was used to assess the relationship between farmers’ ranking and laboratory analysis in evaluating wheat bran quality parameters. The statistical significance was declared at p<0.05.

## RESULTS

**Figure 1:**
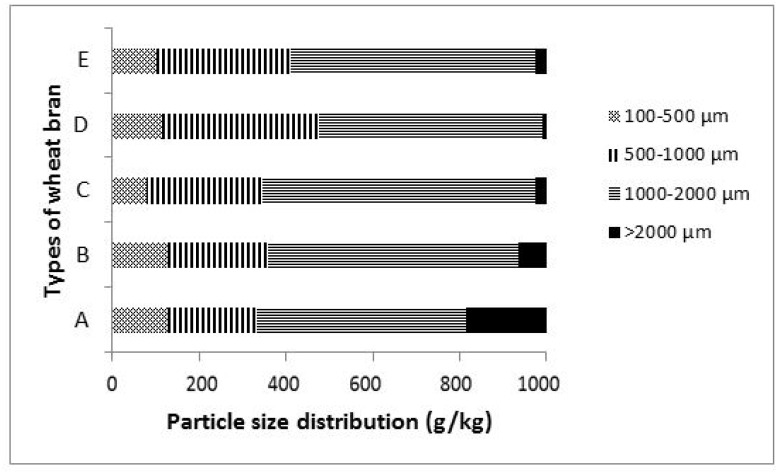
Particle size distribution (g/kg) of wheat bran produced from five major flour factories in Tigray region (Northern Ethiopia).

The particle size distribution of wheat brans is presented in Figure 1. Wheat bran types D and E exhibited predominantly smaller particles (<1 mm), constituting 475 and 412 g/kg, respectively. Conversely, wheat bran types A, B, and C contained mainly larger particles (>1 mm), ranging from 640 to 668 g/kg. Wheat bran type C had lower BD but higher WHC as compared to other wheat bran types (Table 1). The most pronounced variation in particle size distribution (>2 mm) was observed in wheat bran type A, which had the largest fraction (180 g/kg) of the most coarse material compared to other wheat bran types (Figure 1).

Although farmers rated wheat bran types D and E lower for WHC (p<0.001) and suitability for digestive function (p<0.001) compared to wheat bran types A, B, and C, wheat bran types D and E had a higher overall RI (Table 2). As indicated in Table 3, wheat bran particle size was positively correlated with WHC (r_spearman’s_ = 0.526; p*<*0.05; N = 30) and suitability for digestive function (lower digestive disorder risk, r_spearman’s_ = 0.646; p<0.05; N = 30). Further, farmers’ scores for nutritive value correlated positively with CP (r_spearman’s_ = 0.347; p<0.05; N = 30) and effective rumen degradability of crude protein (ERDCP) (r_spearman’s_ = 0.291; p<0.05; N = 30), and negatively with particle size (r_spearman’s_ = −0.553; p<0.05; N = 30).

## DISCUSSION

Wheat bran, a milling by-product containing the pericarp, seed coats, nucellus, and aleurone cells [14], is not a standardized product with defined quality and chemical composition [15]. It represents 11% [16] and up to 26% [1] of total wheat grain weight, depending on the milling process. Grinding conditions such as mill type, screen size, mill speed, and equipment condition affect particle size distribution [14]. Considerable variations in average particle size and distribution were observed among wheat bran types (Table 1). A lower geometric standard deviation (S_gw_) indicated greater uniformity of the particle size [7]. Wheat bran types with a smaller D50 show more uniformity, while the wheat brans with larger D50 show less uniformity. Type A wheat bran had a higher S_gw_, indicating a broader particle size distribution (Figure 1).

Coarse wheat bran exhibits better hydration properties (WHC and SC) compared to finer bran [3,4,17]. Specifically, Auffret et al [17] noted a twofold increase in SC when particle size increased from 320 to 900 μm, while Jacobs et al [3] reported a threefold increase for sizes from 78 to 1,687 μm. However, the relationship between D50 and WHC was weak (r_pearson_ = 0.355, p = 0.555, N = 5; data not shown) in the current study, likely due to the limited number of samples and narrow particle size range (908 to 1,103 μm), while also factors like BD may influence WHC. Specifically, wheat bran type C with low BD exhibited enhanced hydration properties probably due to increased inter-particle spacing, resulting in a more porous structure [17].

The CP, EE, aNDF_OM_, and ERDCP content of wheat bran was within ranges reported in the Feedipedia feed database [18]. Wheat bran, contains CP ranging from 13.3% to 16%, classifying it as a moderate-protein feed sources (CP content: 10%–16%; Moran [19]). In the present study all the wheat bran types meet the minimum threshold of 125 g ERDCP/kg effective rumen degradability of dry matter (ERDDM) for optimal ruminal microbial yield [20]. From a practical standpoint, wheat bran, with 144–207 g ERDCP/kg ERDDM, is commonly used in dairy farms as a supplement that could supply extra energy while also complementing rumen degradable protein shortages for optimal rumen microbial yield.

Farmers’ perceptions of feed quality, particularly regarding particle size distribution, are primarily shaped by practical experience and observational methods rather than formal laboratory assessments. This informal evaluation relies on empirical knowledge gained from years of livestock feeding and management, such as observing milk yield in cows fed wheat bran with varying particle size distributions, often through visual inspection (personal communications).

Dairy farmers preferred finer wheat bran as compared to the coarse product for its better nutritive value, attributed to perceived higher endosperm-to-bran ratio. Farmers’ qualitative appreciation of the nutritive value correlated moderately with CP (r_spearman’s_ = 0.347, p<0.05) and ERDCP (r_spearman’s_ = 0.291, p<0.05). Moreover, the fine wheat bran types had a higher overall RI (Table 2). This indicates that farmers prioritize nutritive values over other parameters in the ranking wheat bran. Previous studies have shown a strong positive correlation between farmers’ feed value scores and CP content in multipurpose forage trees (r = 0.814; Mekoya et al [5]), as well as moderate correlation in indigenous fodder trees and shrubs (r = 0.32; Yisehak and Janssens [6]). The current study suggests that even for industrial side products like wheat bran, farmers’ qualitative evaluations are valuable due to their practical experience. However, it remains unclear whether these findings are generalizable, as farmers’ expertise relies on locally available products and their specific regional context. Moreover, this explorative study had a small sample size. Further research is warranted with a broader range of particle sizes, a larger sample size and a more diverse set of regional markets to confirm these results. Extension services can provide targeted training on feed assessment, quality parameters, and analysis through participatory approaches that integrate local perceptions of feed quality with scientific evidence, enhancing farmers’ decision-making and productivity. Further, integrating farmers’ experiential insights into research through collaborative platforms—such as farmer-scientist dialogues, field demonstrations, and adaptive trials—can bridge field-based knowledge with scientific feed quality indicators, ensuring their validation within a practical production context.

## CONCLUSION

Particle size distribution is the predominant qualitative selection criteria for farmers to assess wheat bran quality (e.g. on the market) and this qualitative appreciation is to some extent related to chemical characteristics and rumen degradability. Farmers scored coarse wheat bran types for better WHC and lower risks of digestive disorder, while fine wheat bran types were valued for higher nutritive value. The observed variation in physical and nutritive value attributes of the wheat bran produced by the flour processing factories calls for a standardization of this by-product. By combining participatory training with collaborative research, extension services and farmer engagement can bridge field-based knowledge with scientific feed quality indicators, enhancing decision-making, productivity, and practical validation.

## Figures and Tables

**Table 1:** Chemical composition (g/kg DM, unless stated otherwise), effective rumen dry matter and crude protein degradability, and physical parameters of wheat bran produced from five flour processing factories in Tigray region (Northern Ethiopia)

Items	Wheat bran types					SD

	A	B	C	D	E	
Ash	34	37	47	38	42	4.7
Crude protein	144	133	161	152	163	12.4
Fat	39	40	51	43	52	5.8
aNDF_OM_	347	368	476	409	436	48.6
ADF	112	127	157	131	147	17.0
NFC	436	422	265	358	307	73.2
ERDDM (g/g DM)	0.712	0.675	0.568	0.662	0.582	0.0621
ERDCP (g/g CP)	0.747	0.732	0.730	0.793	0.734	0.0264
ERDCP/ERDDM (g/kg)	151	144	207	182	206	29.5
Physical parameters						
D50 (μm)	1,103	1,011	1,046	908	977	79.3
S_gw_	2.05	1.85	1.68	1.78	1.74	0.136
Bulk density (g/mL)	0.36	0.36	0.27	0.36	0.36	0.041
WHC (mL/g)	2.28	2.49	2.90	2.15	2.49	0.277
Swelling capacity (mL/g)	2.00	2.13	2.80	2.13	2.00	0.396

SD, standard deviation; aNDF_OM_, alpha amylase-treated neutral detergent fiber expressed exclusive ash; ADF, acid detergent fiber; NFC, non-fiber carbohydates; ERDDM, effective rumen degradability of dry matter; DM, dry matter; ERDCP, effective rumen degradability of crude protein; CP, crude protein; D50, geometric mean particle size of wheat bran or mesh size of an hypothetical screen which would retain 50% of the particles; S_gw_, geometric standard deviation; WHC, water holding capacity.

**Table 2:** Mean rank values of different wheat bran types evaluated by farmers’ criteria for nutritional and hydration properties as well as risks of digestive disorder parameters

Parameters^1)^	Wheat bran types					CI	p-value

	A	B	C	D	E		
Nutritive value^2)^	2.43^d^	2.77^c^	2.03^d^	3.13^b^	3.97^a^	[2.69, 4.00]	<0.001
WHC^2)^	3.63^b^	3.80^b^	4.00^a^	3.20^c^	2.83^d^	[1.78, 4.00]	<0.001
Risks for digestive disorder^3)^	4.00^a^	4.00^a^	4.00^a^	3.40^b^	2.93^c^	[2.77, 4.00]	<0.001
Ranking index	0.15	0.19	0.12	0.26	0.28	-	-

1) Wilcoxon rank test was performed.

2) The ranking scores for parameters (1 = poor, 2 = fair, 3 = good, 4 = excellent).

3) The ranking scores for parameters (1 = high, 2 = moderate, 3 = low, 4 = very low).

a–d Means in a row with different superscripts are significantly different (p<0.05).

CI, 95% confidence interval; WHC, water holding capacity.

**Table 3:** Spearman’s rank correlation of farmers ranking scores with laboratory parameters for wheat bran quality assessment

Laboratory parameters	Farmers’ ranking scores for wheat bran quality attributes		

	Nutritive value	WHC	Risks for digestive disorder
D50 (μm)	−0.553^*^	0.526^*^	0.646^*^
WHC (mL/g)	−0.196^*^	0.367^*^	0.217^*^
Bulk density (g/mL)	0.463^*^	−0.468^*^	−0.331^*^
Swelling capacity (mL/g)	−0.430^*^	0.476^*^	0.364^*^
CP (g/kg DM)	0.347^*^	−0.377^*^	−0.579^*^
aNDF_OM_ (g/kg DM)	0.092	−0.040	−0.290^*^
NFC (g/kg DM)	−0.092	0.040	0.290^*^
ERDDM (g/g DM)	−0.092	0.040	0.290^*^
ERDCP (g/g CP)	0.291^*^	−0.477^*^	−0.356^*^

* p<0.05.

D50, geometric mean particle size of wheat bran or mesh size of an hypothetical screen which would retain 50% of the particles; WHC, water holding capacity; CP, crude protein; DM, dry matter; aNDF_OM_, alpha amylase-treated neutral detergent fiber expressed exclusive ash; NFC, non-fiber carbohydates; ERDDM, effective rumen degradability of dry matter; ERDCP, effective rumen degradability of crude protein.
